# Low-density lipoprotein (LDL)-dependent uptake of Gram-positive lipoteichoic acid and Gram-negative lipopolysaccharide occurs through LDL receptor

**DOI:** 10.1038/s41598-018-28777-0

**Published:** 2018-07-12

**Authors:** Peter M. Grin, Dhruva J. Dwivedi, Kevin M. Chathely, Bernardo L. Trigatti, Annik Prat, Nabil G. Seidah, Patricia C. Liaw, Alison E. Fox-Robichaud

**Affiliations:** 1 Thrombosis and Atherosclerosis Research Institute, McMaster University, Hamilton, ON Canada; 2 Department of Medicine, McMaster University, Hamilton, ON Canada; 3 Department of Biochemistry and Biomedical Sciences, McMaster University, Hamilton, ON Canada; 4 Laboratory of Biochemical Neuroendocrinology, Institut de Recherches Cliniques de Montréal, University of Montréal, Montréal, QC Canada

## Abstract

Lipoteichoic acid (LTA) and lipopolysaccharide (LPS) are bacterial lipids that stimulate pro-inflammatory cytokine production, thereby exacerbating sepsis pathophysiology. Proprotein convertase subtilisin/kexin type 9 (PCSK9) negatively regulates uptake of cholesterol by downregulating hepatic lipoprotein receptors, including low-density lipoprotein (LDL) receptor (LDLR) and possibly LDLR-related protein-1 (LRP1). PCSK9 also negatively regulates Gram-negative LPS uptake by hepatocytes, however this mechanism is not completely characterized and mechanisms of Gram-positive LTA uptake are unknown. Therefore, our objective was to elucidate the mechanisms through which PCSK9 regulates uptake of LTA and LPS by investigating the roles of lipoproteins and lipoprotein receptors. Here we show that plasma PCSK9 concentrations increase transiently over time in septic and non-septic critically ill patients, with highly similar profiles over 14 days. Using flow cytometry, we demonstrate that PCSK9 negatively regulates LDLR-mediated uptake of LTA and LPS by HepG2 hepatocytes through an LDL-dependent mechanism, whereas LRP1 and high-density lipoprotein do not contribute to this uptake pathway. Bacterial lipid uptake by hepatocytes was not associated with cytokine production or hepatocellular injury. In conclusion, our study characterizes an LDL-dependent and LDLR-mediated bacterial lipid uptake pathway regulated by PCSK9, and provides evidence in support of PCSK9 inhibition as a potential therapeutic strategy for sepsis.

## Introduction

Sepsis is defined as life threatening organ dysfunction caused by a dysregulated host response to infection^1^, which is most commonly bacterial and triggers systemic inflammation. Current treatment of septic patients relies upon fluid resuscitation to stabilize hemodynamics and antibiotics to target the infection^2^. However, mortality rates in septic patients still range from 15% to as high as 50%^3–5^, which suggests that additional treatment strategies are necessary. Novel therapeutic approaches could involve improving clearance of pro-inflammatory bacterial lipids, including lipoteichoic acid (LTA) from Gram-positive bacteria and lipopolysaccharide (LPS)—also known as endotoxin—from Gram-negative bacteria. These bacterial lipids are pathogen-associated molecular patterns (PAMPs) that stimulate the innate immune response through binding to pattern recognition receptors (PRRs), such as Toll-like receptors (TLRs) expressed by monocytes, macrophages, neutrophils, and other immune cell types^6^. Gram-negative LPS mainly signals through TLR4^7^, while Gram-positive LTA can bind to and signal through TLR2^8,9^; both of these interactions stimulate downstream activation of NF-κB^6^, which results in transcription and secretion of multiple pro-inflammatory cytokines that play important roles in inflammatory diseases such as sepsis^10^. Widespread release of these pro-inflammatory cytokines during sepsis drives systemic leukocyte recruitment in the liver and other organs^11–13^. Furthermore, recruitment of activated leukocytes to the hepatic microcirculation during endotoxemia has been correlated with hepatocyte apoptosis^14^, potentially contributing to liver dysfunction. Therefore, reducing cytokine-driven inflammation through improved clearance of bacterial lipids may be a novel therapeutic strategy to ameliorate the pathophysiology of sepsis.

To prevent unwarranted inflammation, the body has several detoxification and clearance mechanisms for bacterial lipids. For example, LPS binds to various lipid transfer proteins within the blood^15–17^, which can facilitate detoxification either directly^15^ or indirectly through transfer into lipoproteins^16,18^. The latter attenuates the biological activity of LPS by sequestering the lipid A region within the phospholipid membrane of the lipoprotein^16,17,19^. The uptake and clearance mechanisms of bacterial lipids are much less clear than transfer and detoxification mechanisms, despite some evidence suggesting a role for the hepatobiliary route of excretion for lipoprotein-bound LPS in rats^20^. Both LPS and LTA are known to distribute into low-density lipoprotein (LDL) and high-density lipoprotein (HDL)^21,22^, suggesting that similar pathways may be involved in handling both types of bacterial lipids; however, to our knowledge there are no reports of lipoprotein-dependent uptake pathways for both Gram-positive and Gram-negative bacterial lipids. Further understanding of the role of lipoproteins in regulating bacterial lipid uptake and clearance may lead to new approaches for therapeutic modulation of the physiologic response to infection, and could help to improve our understanding of sepsis pathophysiology.

Proprotein convertase subtilisin/kexin type 9 (PCSK9) is a key negative regulator of hepatic lipoprotein receptors, including low-density lipoprotein (LDL) receptor (LDLR)^23^ and possibly LDLR-related protein 1 (LRP1)^24^, thereby regulating lipid metabolism and homeostasis^25–27^. PCSK9 deficiency or inhibition has been associated with reduced plasma cytokine levels and improved survival in both septic shock patients and mouse models of sepsis^28,29^. Our recent studies also demonstrate that PCSK9 overexpression exacerbates sepsis pathophysiology through increased inflammation in the lungs and liver, whereas PCSK9 deficiency reduces the infectious burden, lung inflammation, and hepatocellular injury in septic mice^29^. Therefore, PCSK9 may be a novel therapeutic target for treatment of sepsis, particularly once its mechanistic role in sepsis is elucidated.

PCSK9 is known to reduce uptake of LPS by hepatocytes^28,30^, presumably through downregulation of LDLR, although the precise mechanism has not been directly demonstrated and it is unknown if hepatic LRP1 is also involved in such uptake. Previous studies have shown that LDLR can clear LPS from the circulation^30^, but the underlying mechanisms are unclear. Furthermore, to our knowledge the mechanisms of LTA uptake by hepatocytes have not been studied, and therefore it is unknown whether PCSK9-regulated hepatocyte clearance of bacterial lipids is restricted to Gram-negative LPS. Since both LPS and LTA are known to incorporate into LDL^21,22^, and LDL is the classical ligand for LDLR^31^, we hypothesized that PCSK9 negatively regulates LDLR-mediated uptake of both Gram-negative LPS and Gram-positive LTA, and that such uptake is LDL-dependent. We also investigated the potential involvement of LRP1 and HDL in bacterial lipid uptake by human HepG2 hepatocytes, and assessed the physiologic response to varying degrees of bacterial lipid uptake by these cells. Finally, we measured PCSK9 concentrations in the plasma of critically ill septic and non-septic patients over time to assess the clinical relevance of PCSK9 in sepsis.

## Results

### Plasma PCSK9 concentrations in ICU patients with sepsis

**Figure 1:**
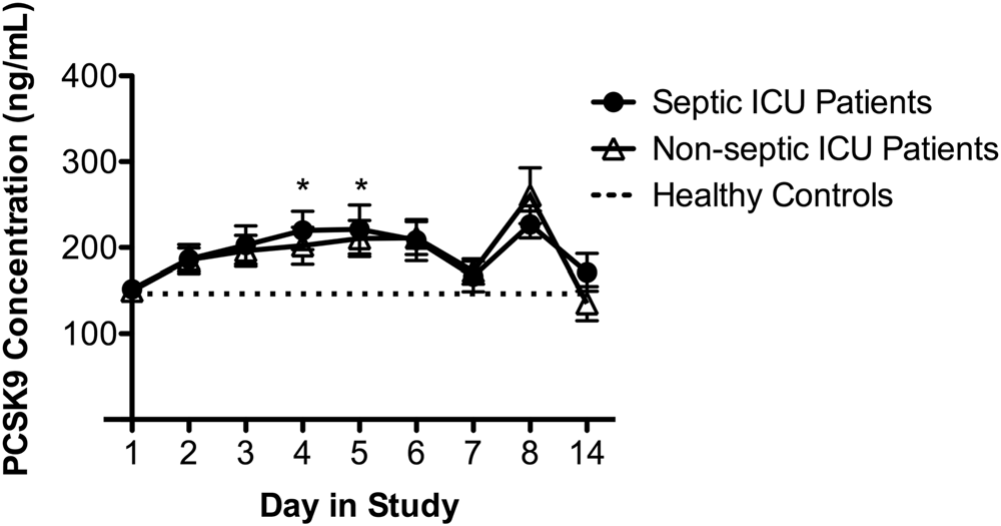
Plasma PCSK9 concentrations in septic and non-septic ICU patients over time compared to healthy controls. Plasma for healthy controls was collected on a single day (mean PCSK9 concentrations of 146.4 ± 13.4 ng/mL). Data are expressed as mean ± SEM, *p < 0.05 vs. healthy control by two-way ANOVA.

To determine whether PCSK9 levels are affected by sepsis in critically ill patients, we measured plasma PCSK9 concentrations over time in intensive care unit (ICU) patients with sepsis or septic shock from the DYNAMICS study. Baseline (Day 1) PCSK9 concentrations in both septic and non-septic ICU patients were comparable to those in healthy controls, then increased over the first 5 days in the study, and returned to normal levels by Day 7 (Fig. 1). Peak plasma PCSK9 concentrations were observed at Days 4 & 5, and these concentrations were significantly greater than plasma PCSK9 concentrations measured in healthy volunteers. Data obtained after Day 7 suggests that plasma PCSK9 concentrations continue to transiently increase and return to normal levels during the course of 14 days in ICU, however the Day 8 differences did not reach statistical significance due to low sample size at this time-point. The profile of PCSK9 concentrations over time was strikingly similar in septic and non-septic critically ill patients, demonstrating that elevations in PCSK9 concentrations due to critical illness are not specific to sepsis.

### Receptor targets of PCSK9 involved in LTA & LPS Uptake

**Figure 2:**
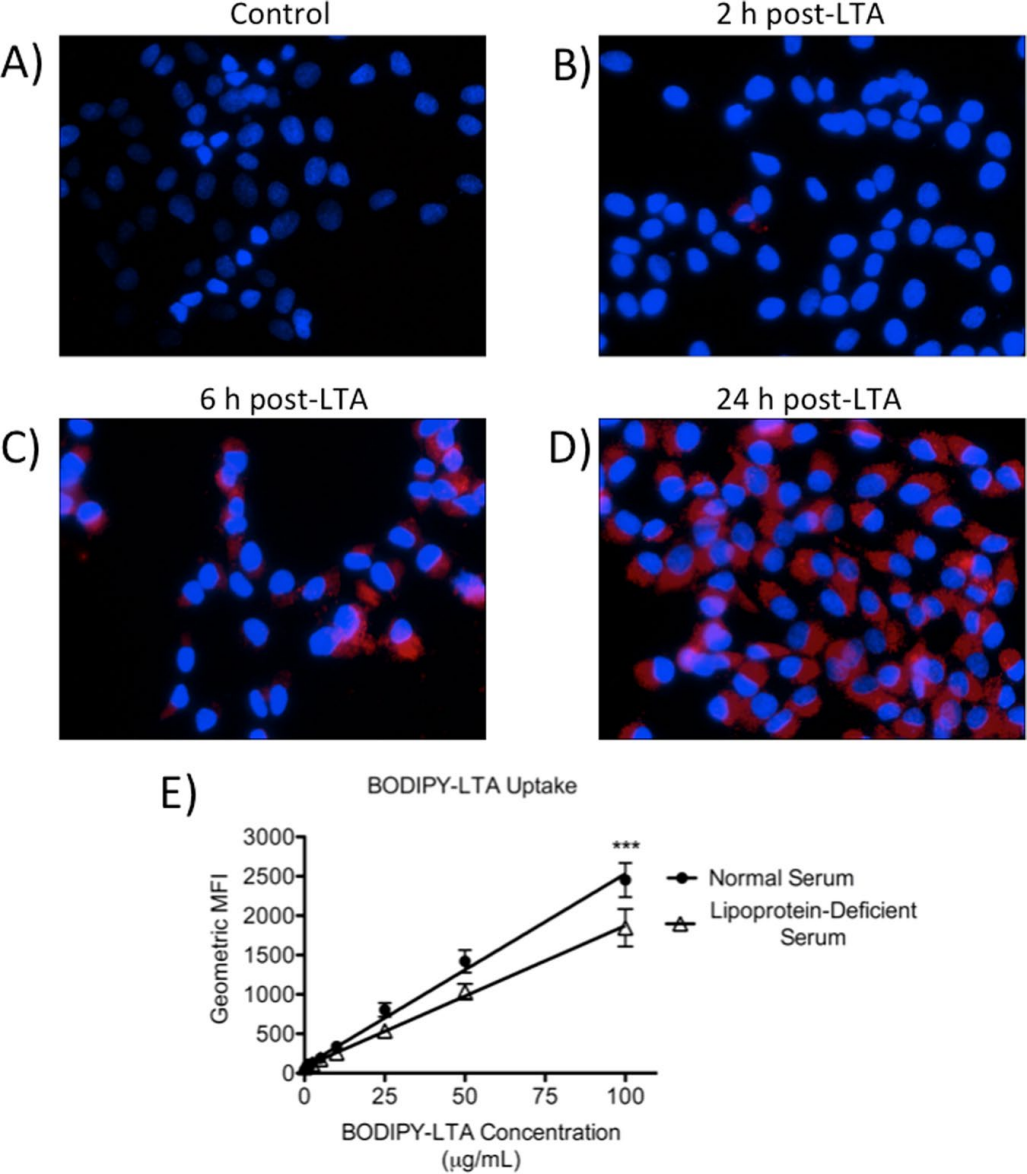
Time-course and dose-response of LTA uptake by HepG2 cells over 24 h. Uptake of fluorescent LTA by cells cultured in 20% normal human serum was visualized through fluorescence microscopy in untreated cells (**A**) or in cells treated with 10 μg/mL of BODIPY 630/650-LTA for 2 h (**B**), 6 h (**C**), or 24 h (**D**), or measured via flow cytometry for cells cultured in either 20% normal serum or lipoprotein-deficient serum (prepared in laboratory) and treated with increasing concentrations of fluorescent LTA over 24 h (**E**). Nuclei are stained in blue with DAPI, and BODIPY 630/650-LTA appears red (200× magnification). Data are representative of 3 experiments (**A**–**D**), and are expressed as mean ± SEM from 3 experiments (**E**). ***p < 0.001 by two-way ANOVA. MFI, mean fluorescence intensity.

To assess whether LTA uptake by hepatocytes occurs similarly to previously documented LPS uptake^30^, we first examined the time- and dose-dependence of this process in human HepG2 hepatocytes, which express many of the same receptors as primary hepatocytes, including LDLR and LRP1^24^. Over the course of 24 h, HepG2 cells progressively internalized BODIPY-LTA in a time-dependent (Fig. 2A–D) and dose-dependent manner that is partly lipoprotein-dependent (Fig. 2E) and TLR-2 independent (see Supplementary Fig. S1). Cell surface binding of LTA was observed in conjunction with internalization (hereafter referred to as uptake) using confocal microscopy to dynamically image live HepG2 cells *in vitro* following LTA treatment; the fluorescence intensity from uptake was notably greater than intensity from cell surface binding even by 3 h post-treatment, and further increased over time up to 6 h post-treatment (Supplementary Fig. S2; Supplementary Videos S1 and S2).

**Figure 3:**
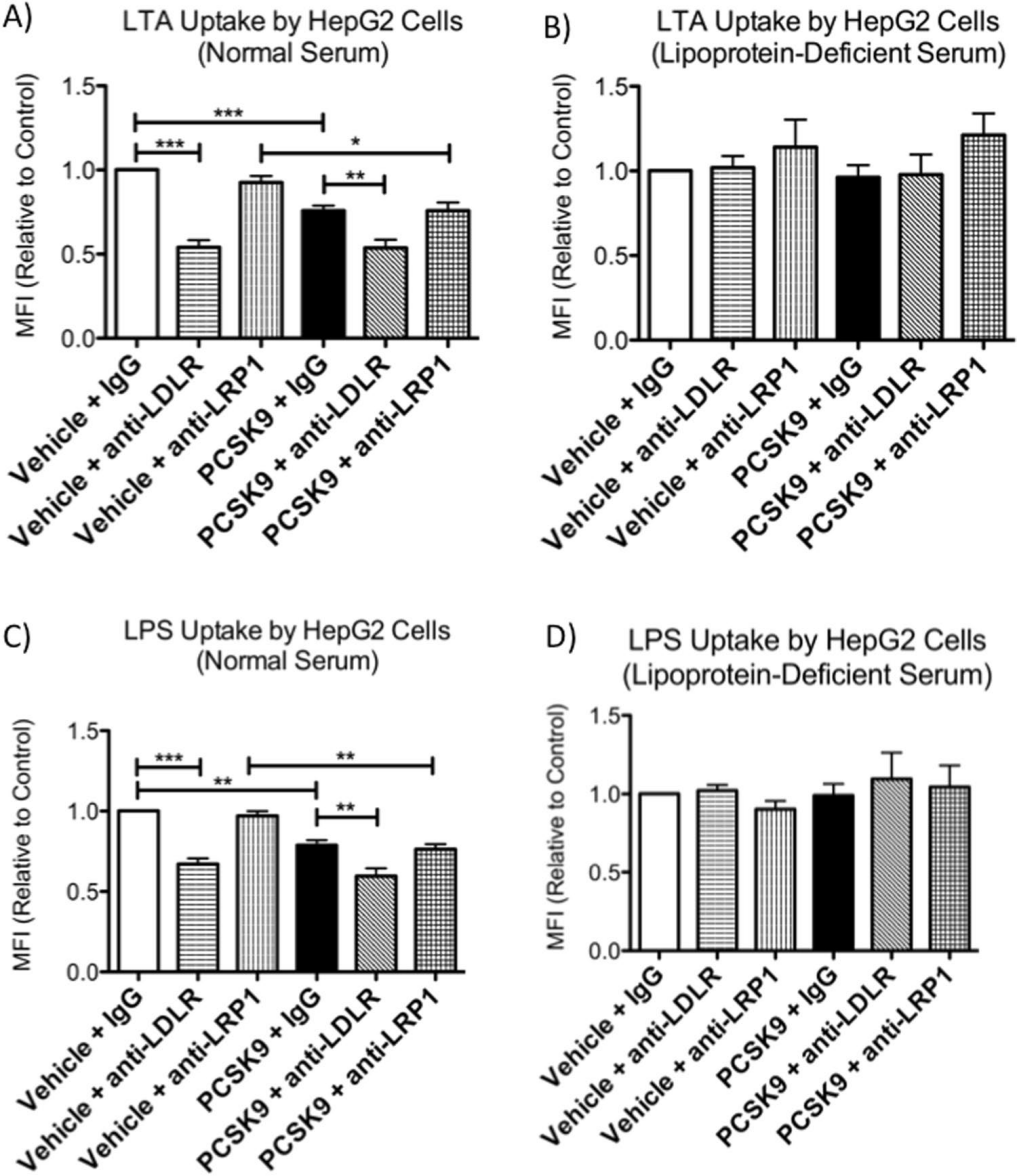
PCSK9 regulates lipoprotein-dependent uptake of LTA and LPS by HepG2 cells through LDLR, not LRP1. HepG2 cells were cultured in 20% normal human serum (**A**,**C**) or (commercially available) lipoprotein-deficient human serum (**B**,**D**) and were pre-treated with 2.5 μg/mL of recombinant human PCSK9 or vehicle control at 6 h before, as well as anti-LDLR, anti-LRP1, or control IgG antibodies at 2 h before treatment with BODIPY 630/650-LTA (10 μg/mL; A,B) or AlexaFluor 488-LPS (2.5 μg/mL; **C**,**D**) for 24 h. Data were collected from 4–5 experiments at 24 hours after LPS or LTA treatment using flow cytometry, and are expressed as geometric mean ± SEM. *p < 0.05, **p < 0.01, ***p < 0.001 by one-way ANOVA. MFI, mean fluorescence intensity.

To investigate which PCSK9-regulated lipoprotein receptors are involved in uptake of bacterial lipids, we treated human HepG2 cells with an LDLR-blocking antibody^32^, an LRP1-blocking antibody^33^, or control IgG, with or without PCSK9 pre-treatment, and measured uptake of fluorescently labeled LTA or LPS. These experiments were conducted in cell culture media containing either 20% normal serum or 20% lipoprotein-deficient serum, in order to further assess whether lipoproteins are required for LPS and LTA uptake pathways that are regulated by PCSK9. For experiments conducted in normal serum, we observed that PCSK9 and anti-LDLR treatments significantly reduced uptake of LTA and LPS compared to controls, while the LRP1-blocking antibody had no effect (Fig. 3A,C). Treating cells with both PCSK9 and the LDLR-blocking antibody did not have an additive effect on uptake of LPS or LTA, which suggests that the negative regulatory effects of PCSK9 on bacterial lipid uptake by HepG2 cells occur primarily through LDLR and not through other receptors in this cell type. This mechanism was also found to be lipoprotein-dependent, as PCSK9 and anti-LDLR treatments had no effect on LTA or LPS uptake by HepG2 cells when the cells were cultured in medium containing lipoprotein-deficient serum (Fig. 3B,D). TLR2 or TLR4 did not contribute to LTA or LPS uptake by HepG2 cells, as anti-TLR4 and anti-TLR2 antibodies resulted in dose-dependent increases of LPS and LTA uptake, respectively (Supplementary Fig. S1). Collectively, these findings demonstrate that PCSK9 regulates lipoprotein-dependent bacterial lipid uptake through LDLR, not through LRP1, and that bacterial lipid uptake mechanisms are TLR2- and TLR4-independent.

### Physiological Response of HepG2 Cells to Variable Uptake of LPS and LTA

In order to assess if increased uptake of LPS or LTA results in an inflammatory response or injury to hepatocytes, we measured inflammatory cytokines including IL-6, IL-8, IL-10, and IL-17, as well as alanine aminotransferase (ALT) activity in the cell culture supernatant obtained from PCSK9-treatment experiments conducted in normal serum. HepG2 cells did not produce IL-6 or IL-8 in response to LPS or LTA, whereas THP-1 monocytes (as a positive control) produced significantly greater concentrations of IL-6 in response to LPS, and IL-8 in response to either LPS or LTA (Supplementary Fig. S3). Neither LPS, LTA, nor other treatments resulted in hepatocellular injury as measured by extracellular ALT activity in the HepG2 cell culture supernatant (Supplementary Fig. S3). Taken together, these findings suggest that neither LPS nor LTA induce a pro-inflammatory response or hepatocellular injury in HepG2 cells.

### LDL is Required for LDLR-mediated Uptake of Bacterial Lipids

**Figure 4:**
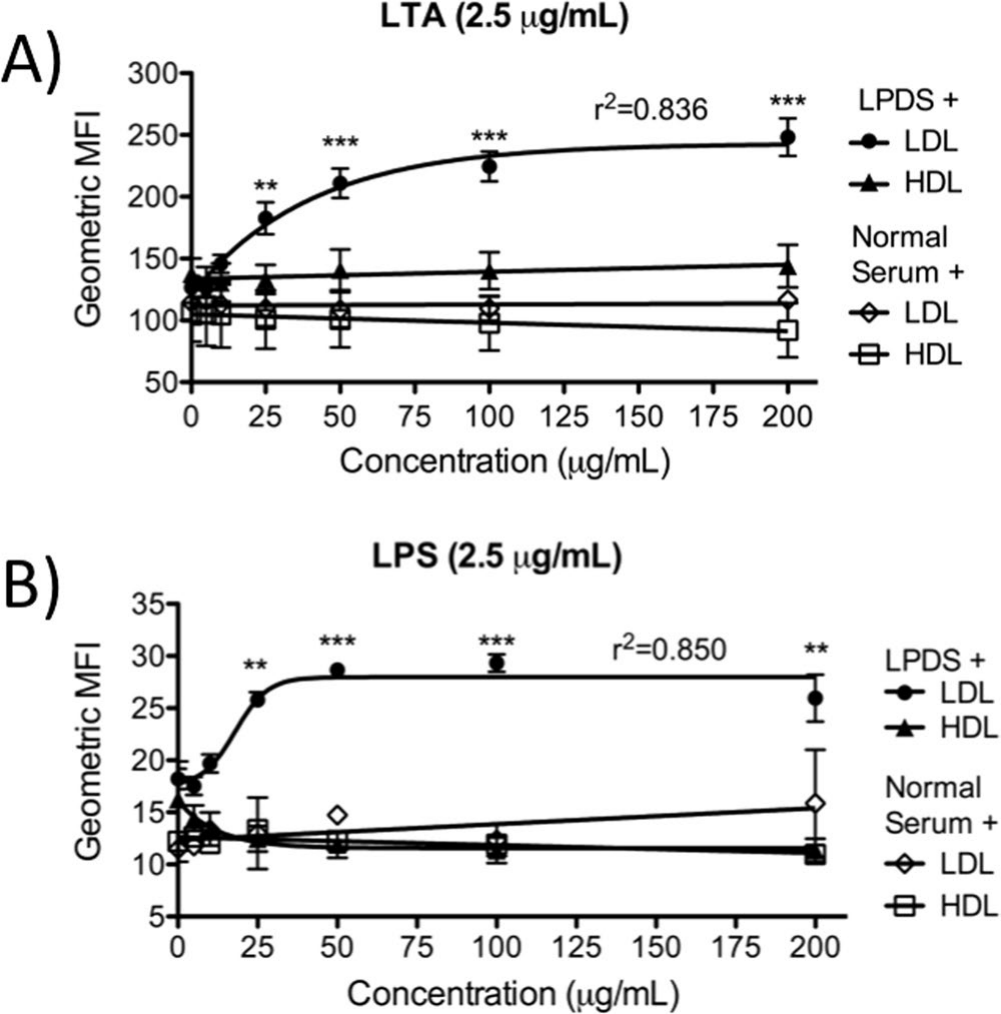
Dose-response of LDL or HDL add-back to normal or lipoprotein-deficient serum on the uptake of LTA and LPS by HepG2 cells. Cells were cultured in 20% lipoprotein-deficient serum (prepared in laboratory) and treated with 2.5 μg/mL of BODIPY 630/650-LTA (**A**) or 2.5 μg/mL of AlexaFluor 488-LPS (**B**) over 24 h in the presence of increasing concentrations of LDL or HDL. Data are shown as mean ± SEM from 3–4 experiments. **p < 0.01, ***p < 0.001 vs. 0 μg/mL of LDL by one-way ANOVA. LPDS, lipoprotein-deficient serum; MFI, mean fluorescence intensity.

To determine which lipoproteins are required for LDLR-mediated uptake of LPS and LTA by HepG2 cells, we first performed dose-response experiments to assess the effects of increasing LDL or HDL concentrations on bacterial lipid uptake. Adding LDL back into lipoprotein-deficient serum, but not normal serum, at increasing concentrations resulted in saturable dose-dependent increases in LTA and LPS uptake by HepG2 cells, whereas add-back of HDL demonstrated no such effect (Fig. 4A,B). These data suggest that LDL may be required for LDLR-mediated uptake of bacterial lipids, while HDL is not, and that LDL concentrations are a limiting factor in determining the extent of bacterial lipid uptake by HepG2 cells.

**Figure 5:**
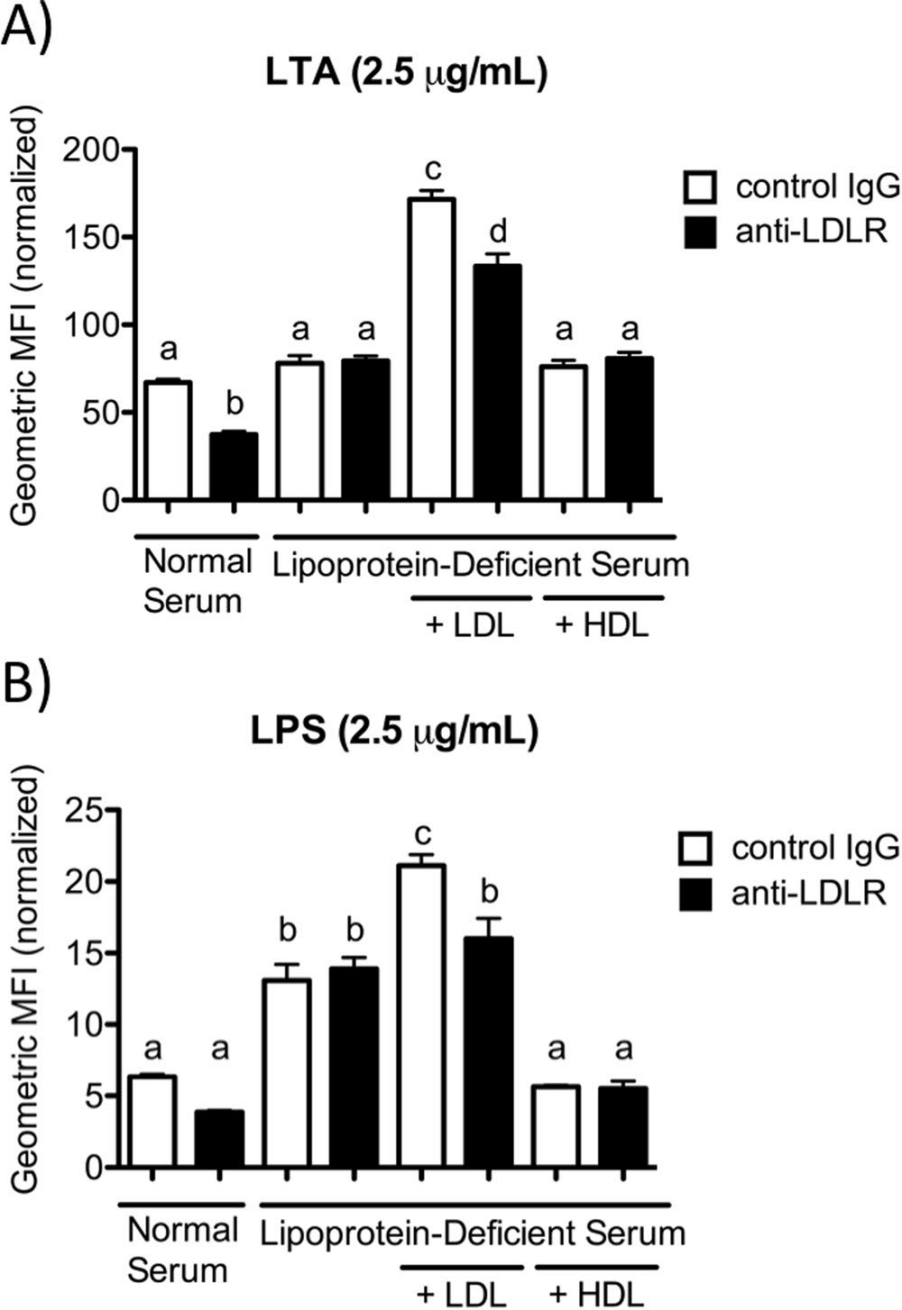
LDL-dependent uptake of LTA and LPS by HepG2 cells is mediated by LDLR. Cells were cultured in 20% normal human serum or donor-matched lipoprotein-deficient serum (prepared in laboratory), with or without 100 μg/mL of LDL or HDL, and treated with anti-LDLR antibody or control IgG for two hours prior to 24 h treatment with 2.5 μg/mL of BODIPY 630/650-LTA (**A**) or AlexaFluor 488-LPS (**B**). Data are presented as geometric mean fluorescence intensity ± SEM from 3–4 experiments, and were normalized by subtracting background auto-fluorescence of untreated cells. Different letters indicate significant differences between groups with p < 0.01 by ANOVA. MFI, mean fluorescence intensity.

To confirm whether the effects of LDL on bacterial lipid uptake occur through LDLR, we performed experiments involving add-back of LDL or HDL to lipoprotein-deficient serum with or without blockade of the LDLR. Consistent with previous experiments, blocking LDLR significantly decreased BODIPY-LTA uptake when cells were cultured in 20% normal serum, but not in lipoprotein-deficient serum (Fig. 5A). Furthermore, addition of 100 μg/mL of LDL to lipoprotein-deficient serum increased LTA uptake compared to lipoprotein-deficient serum alone, and this effect was attenuated by anti-LDLR treatment. On the contrary, addition of 100 μg/mL of HDL to lipoprotein-deficient serum had no effect on BODIPY-LTA uptake compared to normal serum or lipoprotein-deficient serum alone, irrespective of whether LDLR was blocked or not (Fig. 5A). Furthermore, the lack of differences in LTA uptake in normal serum and lipoprotein-deficient serum alone suggests that there are also lipoprotein-independent mechanisms of LTA uptake, which is consistent with findings in Fig. 2E and Supplementary Fig. S1, and these differences are not related to lipopolysaccharide-binding protein concentrations which were similar in normal serum and lipoprotein-deficient serum (Supplementary Fig. S4). Addition of LDL, but not HDL, also increased LPS uptake by HepG2 cells cultured in lipoprotein-deficient serum, and this increase was abolished by treatment with anti-LDLR antibody (Fig. 5B). In these experiments, blocking LDLR also appeared to decrease LPS uptake by HepG2 cells cultured in normal serum, however this difference did not reach statistical significance. Interestingly, HepG2 cells cultured in lipoprotein-deficient serum had significantly increased uptake of LPS compared to cells cultured in normal serum (Fig. 5B), an effect which was not observed with LTA (Fig. 5A). Although seemingly paradoxical, this could be explained by HDL-dependent suppression of LPS uptake by HepG2 cells, a phenomenon observed in both Fig. 4B and Fig. 5B. Further differences between LPS and LTA uptake were evident through partial competitive inhibition of LPS uptake, but not LTA uptake, at 10-fold and 100-fold excess of unlabeled bacterial lipid (see Supplementary Fig. S5). Taken together, these findings demonstrate that LDL, but not HDL, is required for LDLR-mediated uptake of bacterial lipids by HepG2 cells.

## Discussion

In this study we established that LPS and LTA, PAMPs from Gram-negative and Gram-positive bacteria, respectively, are taken up by human HepG2 hepatocytes, in part, through a pathway facilitated by LDL, involving LDLR, and negatively regulated by PCSK9. Our findings are consistent with previous studies, which identified the role of LDLR in LPS clearance by hepatocytes^30^, and we have shown that this role also extends to the uptake of LTA. We further identified that the specific lipoprotein required for LDLR-mediated uptake of bacterial lipids is LDL, since LDL add-back to lipoprotein-deficient serum resulted in dose-dependent increases in uptake of both LPS and LTA, while blocking LDLR attenuated this effect. Moreover, LDL add-back to lipoprotein-deficient serum at increasing doses resulted in saturable increases in bacterial lipid uptake, whereas LTA uptake in normal serum did not saturate at concentrations up to 100 μg/mL of LTA, and was not competitively inhibited by up to 100-fold excess of unlabeled LTA, which collectively suggests that LDL in normal serum acts as a sink for LTA similarly to its previously described function as a cholesterol sink^34^. Since increasing LDL concentrations in normal serum did not affect LTA uptake, we hypothesize that LDL-dependent uptake in normal serum may be limited by initial incorporation of LTA into other lipoproteins^22^ thus limiting the rate of transfer into LDL and consequently limiting LDL-dependent uptake of LTA.

We also investigated whether HDL plays a role in bacterial lipid uptake, as previous work has documented that LPS and LTA are transferred to HDL prior to incorporation into LDL in the blood^18,21,22^. Our data indicate that there are differences in the effects of HDL on LPS and LTA uptake—namely that HDL may reduce uptake of LPS, but has no effect on LTA uptake. These findings are somewhat paradoxical as both LPS and LTA are known to incorporate into HDL^21,22^, and studies have shown that HepG2 cells can internalize HDL particles^35^. Furthermore, since LDLR can bind to apoE^36^, which is found on a subset of HDL particles^37^, it was expected that some uptake of HDL and incorporated bacterial lipids might occur through LDLR. Our observation that blocking LDLR has no effect on LPS or LTA uptake in the presence of HDL demonstrates that HDL does not directly contribute to uptake of these bacterial lipids through LDLR, but does not exclude an indirect contribution to the sequestration and/or transfer of bacterial lipids prior to such uptake. Furthermore, HDL may still play a direct role in bacterial lipid uptake by hepatocytes through other receptors that are not regulated by PCSK9, such as scavenger receptor class B type I (SR-BI), which was previously found to play a role in hepatic LPS clearance^38^. Whether SR-BI is involved in LTA uptake has not been studied, thus differences in hepatic SR-BI involvement in the uptake of LPS vs. LTA are possible and may help to explain the minor differences between LPS and LTA uptake that we observed in response to HDL.

Although we cannot exclude the potential contributions of HDL to bacterial lipid sequestration prior to LDLR-mediated bacterial lipid uptake, our data suggest that the role of HDL is minor in comparison to the central role of LDL in this PCSK9-regulated pathway. Importantly, our findings may help to explain the recent observation that PCSK9 inhibition does not improve mortality in a high-dose LPS mouse model of endotoxemia^39^, which contrasts with most other evidence regarding the role of PCSK9 in endotoxemia and sepsis^28–30,40^. Contrary to LDL-rich lipoprotein profiles in humans, HDL is the prevalent lipoprotein found in mouse serum representing ≥70% of total lipoproteins by mass^41,42^, therefore one would expect a minor contribution of the LDL-dependent, LDLR-mediated bacterial lipid uptake pathway in mice, and thus a more modest effect from PCSK9 inhibition in a high-dose endotoxemia model. These fundamental differences in lipoprotein profiles between humans and mice also suggest that the choice of animal model should be carefully considered in future studies aiming to test PCSK9 inhibitors *in vivo* as a therapy for sepsis.

Our findings also suggest the existence of lipoprotein-independent mechanisms of bacterial lipid uptake, which is consistent with previous observations of TLR4-independent LPS uptake mechanisms by hepatocytes^43^. These mechanisms seem to compensate for LDL-dependent uptake mechanisms in the absence of lipoproteins, although this compensation appears to become less effective with increasing concentrations of LTA present (Fig. 2E), whereas our data collectively suggest that LDL-dependent uptake prevails in the presence of normal serum lipoproteins. The presence of multiple different uptake mechanisms suggests that performing bacterial lipid uptake may be a physiological role of hepatocytes, which would not be surprising given the well-known role of hepatocytes in uptake, metabolism and clearance of many drugs and toxins.

Interestingly, variation in the extent of LPS or LTA uptake by human HepG2 hepatocytes did not affect extracellular ALT activity or cytokine concentrations, indicating that this bacterial lipid uptake mechanism does not appear to be toxic to hepatocytes. Studies have shown that HepG2 cells can secrete cytokines in response to LPS from *Salmonella typhimurium*^44,45^, however LPS from different bacterial sources varies in its ability to induce secretion of pro-inflammatory cytokines, including IL-6 and IL-8^46^, and we studied LPS from *Escherichia coli* in order to extend previous observations^28,30^ on the PCSK9-regulated LPS uptake pathway. We speculate that the ability to take up LPS and LTA without stimulating an inflammatory response may be a homeostatic mechanism that allows hepatocytes in the liver to clear bacterial lipids, such as those that regularly translocate into the portal circulation from the gut lumen by crossing the intestinal barrier^47^. Since *E*. *coli* is an abundant bacterial species in the intestinal microbiota^48^, an argument can be made that hepatocytes may have evolved the ability to take up and clear bacterial lipids from such non-pathogenic bacterial species or serotypes that are part of the natural gut microbiota without generating an inflammatory response, whereas pro-inflammatory cytokines could be produced in response to bacterial lipids from pathogenic serotypes or species, such as *S*. *typhimurium*. However, future studies directly comparing cytokine production by hepatocytes in response to uptake of LPS and LTA from pathogenic vs. commensal bacteria would be required to test this hypothesis.

Bacterial lipid uptake by hepatocytes through the pathway we have identified might function to minimize excessive stimulation of immune cell types in the liver, such as liver resident macrophages known as Kupffer cells, which produce cytokines in response to LPS and LTA^49,50^, and during sepsis^51^. It is conceivable that the previously observed reduction of cytokines in the context of PCSK9-deficiency during sepsis^28,29^ may result from increases in the LDLR-mediated uptake of bacterial lipids by hepatocytes, thereby reducing the availability of these bacterial lipid PAMPs to stimulate PRRs on innate immune cells, including Kupffer cells, and consequently reducing cytokine-driven inflammation during sepsis. The importance of cross-talk between hepatocytes and other cell types in the liver during sepsis is well established^52^. Kupffer cells, for example, have long been known to regulate protein synthesis by hepatocytes in response to LPS^53^. Furthermore, processing of LPS by Kupffer cells can improve its binding to hepatocytes while decreasing the pro-inflammatory properties of this modified LPS^54^. We postulate that the uptake of bacterial lipids by hepatocytes can conversely influence the physiological response of Kupffer cells, given this documented cross-talk in response to LPS. Future studies should aim to determine whether Kupffer cells are the main cell type with reduced cytokine secretion in response to increased bacterial lipid uptake by hepatocytes, in order to establish a stronger functional mechanism linking this PCSK9-regulated pathway to the pathophysiology of sepsis.

Regarding clinical significance, our study is the first to measure circulating PCSK9 concentrations over time in septic and non-septic ICU patients, and our findings complement those of Boyd *et al*., which documented increases in PCSK9 during early sepsis in patients from the Emergency Department^40^. Such increases in plasma PCSK9 concentrations of patients with sepsis were correlated with increased odds of developing more than one organ failure^40^. Furthermore, patients with respiratory failure or septic shock had increased PCSK9 levels compared to septic patients without these complications^40^. While Boyd *et al*. measured PCSK9 concentrations in patients from the Emergency Department with early sepsis^40^, our cohort of patients is characterized by more advanced stages of sepsis that result in critical illness. Importantly, our findings indicate that increases in PCSK9 concentration are not specific to septic patients in the context of critical illness. These studies collectively highlight the importance of PCSK9 in critical illness and sepsis pathophysiology, and provide a rationale for targeting PCSK9 to develop novel therapies that could benefit critically ill patients.

Our work has not only elucidated a bacterial lipid uptake pathway with potential therapeutic relevance for sepsis, but also provides insights for improving our understanding of the complex pathophysiology of sepsis, especially in the context of dyslipidemia. Dyslipidemia may be related to clinical outcomes of patients with sepsis: one observational study found reductions in LDL, HDL, and HDL-associated apolipoproteins in sepsis non-survivors compared to survivors^55^, and a recent study found that low LDL-cholesterol levels were associated with increased rates of community-acquired sepsis^56^. These findings suggest that a reduced ability to maintain lipid homeostasis in the setting of reduced circulating LDL levels may increase the risk and/or severity of sepsis, which could be linked to a reduced ability to clear bacterial lipids through the LDL-dependent pathway that we characterized in this study.

In conclusion, our study directly demonstrates the regulatory role of PCSK9 in bacterial lipid uptake and characterizes the pathway by which this occurs via LDLR through an LDL-dependent mechanism, thereby strengthening the evidence in support of important roles for PCSK9, LDLR and LDL in sepsis. These findings also advance our understanding of sepsis pathophysiology as it relates to dyslipidemia, and highlight another important role for hepatocytes in sepsis. The implications of our study are that inhibition of PCSK9 should be further considered as a novel therapeutic strategy for sepsis, and future research should focus on understanding the systemic effects and clinical outcomes of PCSK9 inhibition in patients with sepsis.

## Methods

### Plasma PCSK9 Concentrations in Septic ICU Patients

PCSK9 concentrations were measured in plasma collected from septic and non-septic ICU patients who were enrolled in the DYNAMICS study (DNA as a prognostic marker in ICU patients study, ClinicalTrials.gov identifier: NCT01355042); written informed consent was obtained (in accordance with standard ICU practice) from patients’ substitute decision makers/legally authorized representatives to collect and use blood samples from enrolled patients for research purposes, and all research methods were performed in accordance with relevant guidelines and regulations covered by ethics approval from the Hamilton Integrated Research Ethics Board (McMaster University & Hamilton Health Sciences) and the Research Ethics Boards of all centers participating in DYNAMICS. Septic ICU patients were recruited according to previously described inclusion and exclusion criteria^57^, and non-septic patients were classified as patients with non-septic shock, patients with multiple trauma & shock, or as critically ill in general (detailed inclusion & exclusion criteria are available at ClinicalTrials.gov, NCT01355042); all patients were enrolled between November 2010 and January 2013 from nine tertiary care centers across Canada. PCSK9 concentrations were measured, using ELISA as previously described^58^, in plasma samples from 49 septic patients with documented Gram-positive or Gram-negative bacterial sepsis, in 30 age-matched non-septic ICU patients, and in plasma collected from 14 healthy volunteers.

### Preparation of BODIPY 630/650-LTA and Lipoprotein-Deficient Human Serum

LTA from *Enterococcus hirae* was fluorescently labeled using the BODIPY 630/650-X NHS ester according to manufacturer’s protocols (Life Technologies, Burlington, ON, Canada). BODIPY-LTA was separated from unbound fluorophore by gel filtration chromatography using a Sephadex G25 column, and the absorbance of each eluted fraction was measured by spectrophotometry at the peak excitation wavelength of 632 nm. Concentrated fractions were pooled, and absorbance was measured to calculate the final concentration of fluorescently labeled LTA using the 100,000 cm^−1^ M^−1^ extinction coefficient of the fluorophore.

Lipoprotein-deficient serum was prepared through ultracentrifugation of pooled normal human serum, collected from seven healthy donors, at 298,000 × g for 48 h at 4 °C, after increasing serum density to 1.215 g/mL with KBr as previously described^59^. Following extraction of the lipoprotein-deficient serum fraction, dialysis was performed nine times using 4 L of normal saline (0.9% NaCl) over a total of 96 hours at 4 °C through a SpectraPor 4 dialysis membrane to remove any residual KBr. Total protein concentration in the lipoprotein-deficient serum was measured with a Pierce BCA Protein Assay (Thermo Fisher Scientific, Burlington, ON, Canada), and sterile normal saline was used to adjust the protein concentration to 70 mg/mL.

### HepG2 Cell Culture & LTA Uptake Time Course and Dose-Response

Human HepG2 hepatocellular carcinoma cells (ATCC, Manassas, VA, USA) were cultured in DMEM containing 10% FBS (v/v), and 1% penicillin-streptomycin (v/v; stock concentrations: 100 U/mL of penicillin and 100 μg/mL of streptomycin). Time course experiments of LTA uptake by HepG2 cells were performed by seeding 3 × 10^5^ cells per well into 6-well plates containing sterilized coverslips, and treating cells with 10 μg/mL of BODIPY-LTA for 0, 2, 6, or 24 hours in media containing 80% DMEM (with 1% penicillin-streptomycin) and 20% normal human serum (v/v). Cells were then fixed with 4% paraformaldehyde and the coverslips were mounted with DAPI-containing medium onto microscope slides for imaging. Dose-response experiments were performed in 24-well plates by treating HepG2 cells with 1, 2.5, 5, 10, 25, 50, or 100 μg/mL of BODIPY-LTA over 24 hours in media containing 80% DMEM (with 1% Penicillin-Streptomycin) and 20% normal human serum or donor-matched lipoprotein-deficient human serum prepared in laboratory. Cells were harvested with trypsin/EDTA, washed with sterile PBS, and a BD FACSCalibur Flow Cytometer was used to measure the geometric mean fluorescence intensity of 10,000 cells per treatment group using CellQuest Pro software (BD Biosciences, Franklin Lakes, NJ, USA).

### Effects of PCSK9, and anti-LDLR or anti-LRP1 antibodies on LPS and LTA Uptake

HepG2 cells were seeded overnight into 24-well plates at a density of 5 × 10^4^ cells per well, after which media was changed to 80% DMEM (containing 1% penicillin-streptomycin), and 20% normal human serum or commercially available lipoprotein-deficient human serum (EMD Millipore, Etobicoke, ON, Canada). Cells were then pretreated with 2.5 μg/mL of recombinant human PCSK9 (AcroBiosystems, Cambridge, MA, USA) or vehicle control at 6 hours before LPS or LTA treatment. Two hours prior to LPS or LTA treatment, cells were pre-treated with 5 μg/mL of anti-human LDLR antibody (AF2148; R&D Systems, Minneapolis, MN, USA), anti-human LRP1 antibody (MA1-27198; Thermo Fisher Scientific, Burlington, ON, Canada), or control IgG (Jackson ImmunoResearch Laboratories, West Grove, PA, USA). Cells were then treated with 2.5 μg/mL of AlexaFluor 488-labeled *E*. *coli* O55:B5 LPS of smooth form (Life Technologies, Burlington, ON, Canada) or control non-fluorescent *E*. *coli* O55:B5 LPS (Sigma Aldrich, Oakville, ON, Canada), or 10 μg/mL of BODIPY 630/650-labeled *E*. *hirae* LTA or control non-fluorescent *E*. *hirae* LTA (Sigma Aldrich, Oakville, ON, Canada) for 24 hours. Cells were harvested with trypsin/EDTA, and the geometric mean fluorescence intensity of 10,000 cells per treatment group was measured through flow cytometry with results normalized to controls. Cell culture supernatant was collected from these experiments and frozen at −80 °C for Luminex cytokine assays to measure IL-6, IL-8, IL-10, and IL-17 (R&D Systems, Minneapolis, MN, USA), and for measurement of extracellular ALT activity (Cayman Chemical Company, Ann Arbor, MI). To obtain HepG2 cell lysate as a positive control for ALT activity assays, HepG2 cells were grown to confluence in 24-well plates and lysed with RIPA buffer containing 1% protease inhibitor cocktail at 4 °C for 1 h on a plate shaker, after which cell lysates were cleared by centrifugation at 15,000 × g for 12 min. THP-1 monocytes (ATCC, Manassas, VA, USA) were cultured in RPMI 1640 complete medium containing 10% FBS, and treated with 2.5 μg/mL of non-fluorescent *E*. *coli* LPS or 10 μg/mL of non-fluorescent *E*. *hirae* LTA for 24 h to obtain conditioned media as a positive control for the Luminex cytokine assay.

### Effects of LDL or HDL on LPS & LTA Uptake by HepG2 Cells

Cells were seeded into 24-well plates at a density of 5 × 10^4^ cells per well and allowed to adhere overnight. On the next day, media was changed to 80% DMEM (containing 1% penicillin-streptomycin) and 20% normal serum or donor-matched lipoprotein-deficient human serum prepared in laboratory. Cells were then treated with increasing concentrations of LDL or HDL (0, 5, 10, 25, 50, 100, or 200 μg/mL by protein), and 2.5 μg/mL of AlexaFluor 488-LPS or BODIPY 630/650-LTA for 24 hours. Flow cytometry was used to measure the geometric mean fluorescence intensity of 10,000 cells per group.

### Assessing if LDL-dependent LTA & LPS Uptake is LDLR-mediated

HepG2 cells were seeded into 24-well plates as described previously. The following morning, media was changed to 80% DMEM (enriched with 1% penicillin-streptomycin) and 20% human serum, which was either pooled normal serum (control) from healthy donors, or donor-matched lipoprotein-deficient serum (prepared in laboratory) with or without add-back of 100 μg/mL of purified human LDL or HDL. One hour later, cells were treated with anti-LDLR antibody or control IgG for two hours prior to treatment with 2.5 μg/mL of AlexaFluor-488 labeled LPS or BODIPY 630/650-labeled LTA. After 24 hours, cells were harvested and flow cytometry was performed to assess uptake of the fluorescent bacterial lipids by measuring the geometric mean fluorescence intensity of 10,000 cells per treatment group.

### Statistical Analyses

One-way or two-way analysis of variance (ANOVA), where appropriate, with Bonferroni post hoc tests, were used for all statistical analyses with a significance threshold of p < 0.05 (GraphPad Prism 5.0, La Jolla, CA, USA).

### Data Availability

The datasets generated and analysed during the current study are available from the corresponding author on reasonable request.

## Electronic supplementary material

Supplementary Information

Supplementary Video S1

Supplementary Video S2
