# Supplementary material for: Low-density lipoprotein (LDL)-dependent uptake of Gram-positive lipoteichoic acid and Gram-negative lipopolysaccharide occurs through LDL receptor
Source: Sci Rep. 2018 Jul 12;8:10496. doi: 10.1038/s41598-018-28777-0 (PMC6043579; doi:10.1038/s41598-018-28777-0)
Supplement: Supplementary file 1 — Supplementary Information [file 41598_2018_28777_MOESM1_ESM.docx]

**Low-density lipoprotein (LDL)-dependent uptake of Gram-positive lipoteichoic acid and Gram-negative lipopolysaccharide occurs through LDL receptor**

Peter M. Grin^1^, Dhruva J. Dwivedi^1,2^, Kevin M. Chathely^1^, Bernardo L. Trigatti^1,3^, Annik Prat^4^, Nabil G. Seidah^4^, Patricia C. Liaw^1,2,#^ & Alison E. Fox-Robichaud^1,2,#,^*

**SUPPLEMENTARY INFORMATION**

**
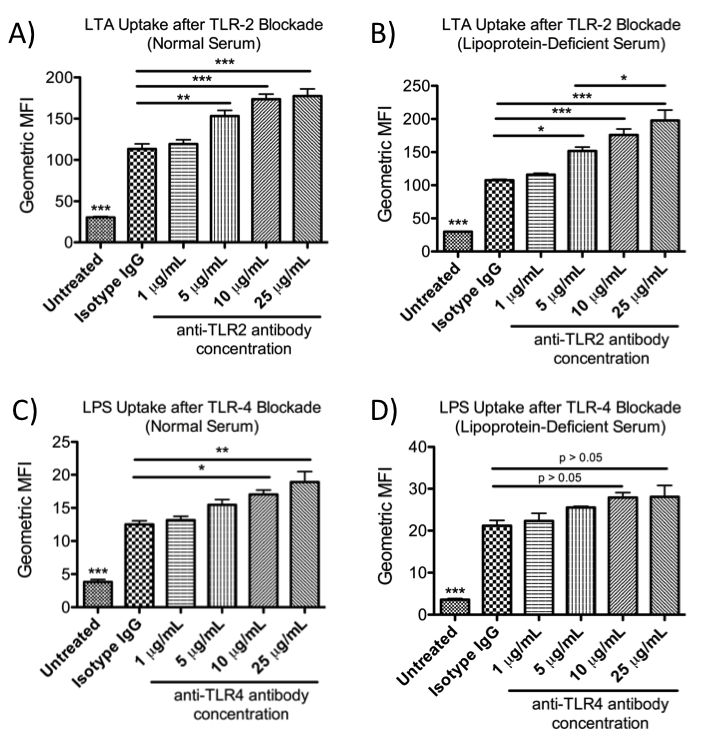
**

**Supplementary Fig. S1. TLR2 or TLR4 blockade dose-dependently increases uptake of LTA or LPS by HepG2 cells, respectively.** Cells were cultured in DMEM containing either 20% normal serum or lipoprotein-deficient serum (prepared in laboratory) and pre-treated with increasing concentrations of anti-mouse/human CD282/TLR2 antibody (clone T2.5; panels A&B) or anti-human CD248/TLR4 antibody (clone HTA125; panels C&D), followed 2 h later by treatment with 2.5 μg/mL of AlexaFluor 488-LPS or BODIPY 630/650-LTA (respectively) for 24 h. Cells were pre-treated with 25 μg/mL mouse IgG isotype control and the same concentrations of fluorescent LPS or LTA to serve as controls. Flow cytometry was performed to quantify uptake of the fluorescently labeled LPS or LTA in 10,000 cells, as measured by geometric mean fluorescence intensity (MFI). Data are expressed as mean ± SEM from 3 independent experiments; *p<0.05, **p<0.01, ***p<0.001.

**
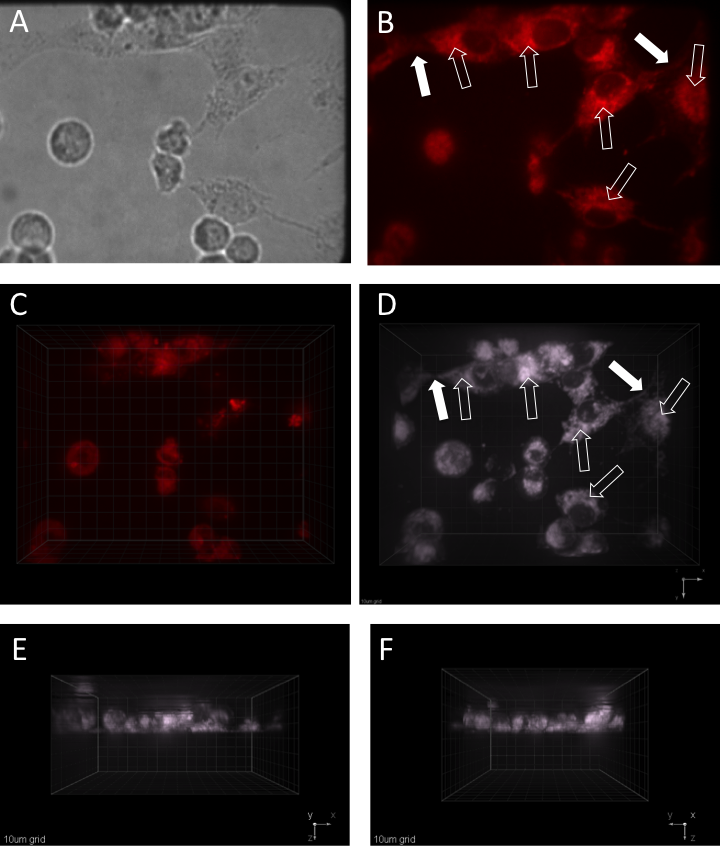
**

**Supplementary Fig. S2. Dynamic live-cell confocal microscopy of LTA uptake by HepG2 cells at 3 h (A-C) and 6 h (D-F) post-treatment demonstrates greater intensity fluorescence from internalization than binding.** (A) Bright-field image of cells at 3 h post-LTA treatment, (B) two-dimensional image of cells binding (solid white arrow) and internalizing (transparent white arrow) LTA at 3 h post-treatment, and (C) three-dimensional top view of reconstructed z-stack at 3 h post-treatment with 10 μg/mL of BODIPY-630/650-LTA. (D-F) are z-stack images of LTA binding (solid arrow) and internalization (transparent arrow) at 6 h post-treatment with 10 μg/mL of BODIPY-630/650-LTA; images in (D-F) are pseudo-coloured in white to enhance contrast of varying degrees of fluorescence. Top (D), front (E), and right (F) views of the three-dimensional z-stack of LTA uptake at 6 h post-treatment. Cells were cultured in DMEM + 20% normal serum and imaged using a 60× water-immersion objective and confocal images were obtained using a Yokagowa CSU22 spinning-disk; data were obtained from 3 independent experiments.


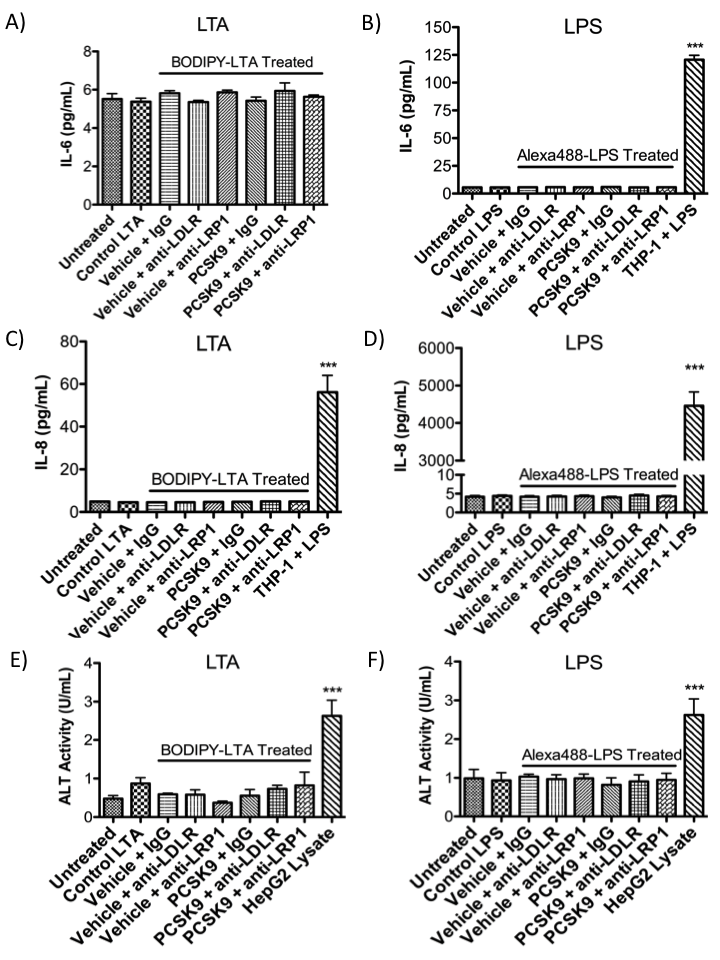


**Supplementary Fig. S3. Cytokine concentrations (A-D) and ALT activity (E,F) in cell culture supernatant collected from HepG2 cells with variable uptake of LTA and LPS following pre-treatment with PCSK9, and/or anti-LDLR or anti-LRP1 antibodies.** HepG2 cells were cultured in 20% normal human serum, and were treated with 10 μg/mL of BODIPY 630/650-LTA (A,C,E) or 2.5 μg/mL of AlexaFluor 488-LPS (B,D,F) for 24 h. THP-1 monocytes were treated over 24 h with 2.5 μg/mL of unlabeled *E. coli* LPS or 10 μg/mL of unlabeled *E. hirae* LTA and cell culture supernatant was collected to serve as a positive control for cytokine secretion. HepG2 lysate was used as a positive control for ALT activity. Data are expressed as mean ± SEM from 4–5 independent experiments. ***p<0.001 by one-way ANOVA. Note: IL-10 and IL-17 concentrations were below the detectable limit, and THP-1 monocytes did not produce IL-6 in response to LTA treatment, thus these data are not shown.


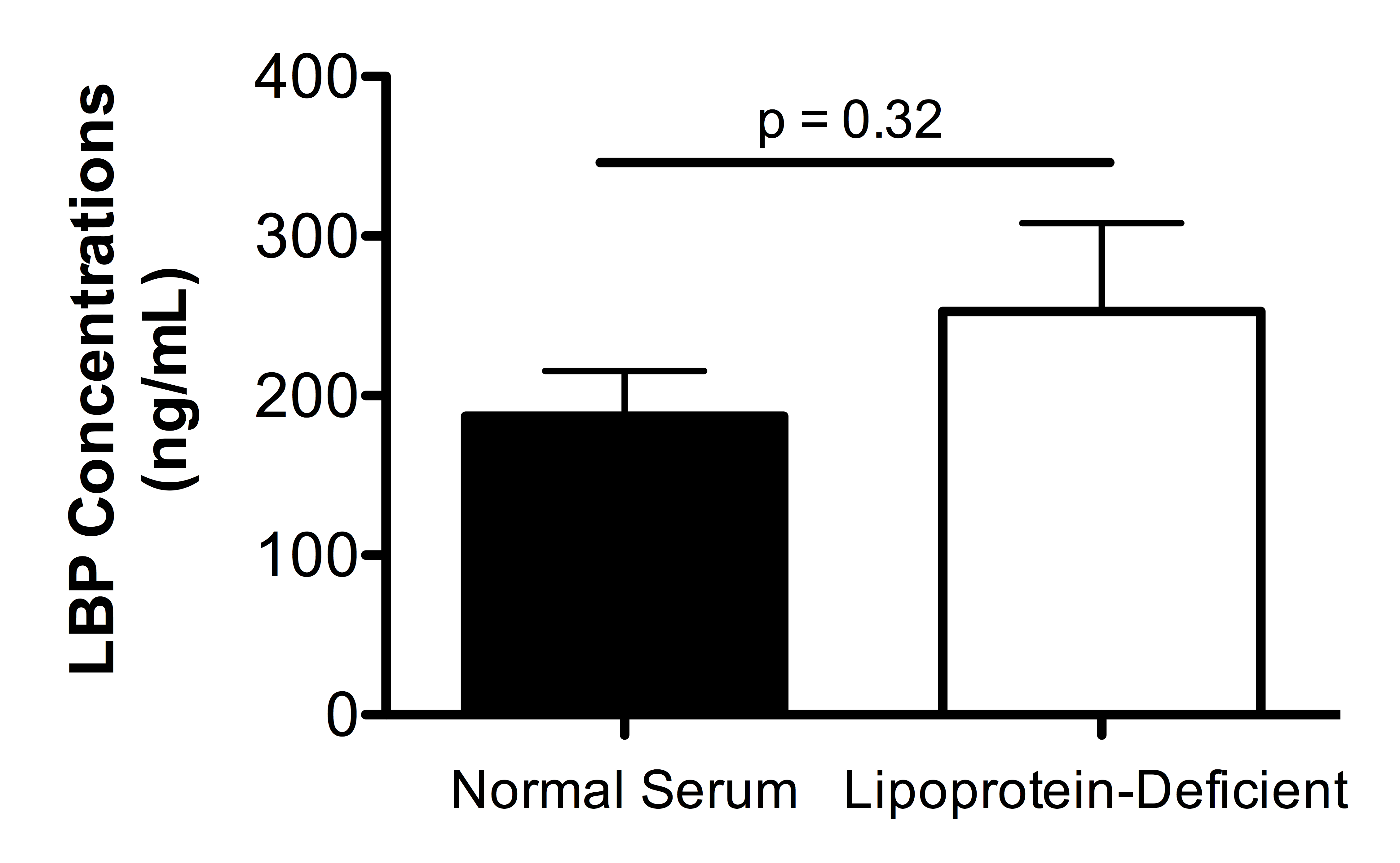


**Supplementary Fig. S4. Lipopolysaccharide-binding protein (LBP) concentrations do not differ significantly between normal serum and donor-matched lipoprotein-deficient serum (prepared in laboratory).** Concentrations were measured using ELISA (R&D Systems). Data are expressed as mean ± SEM; n = 5, from two independent lipoprotein-depletion experiments. p = 0.32 by Student’s t test.


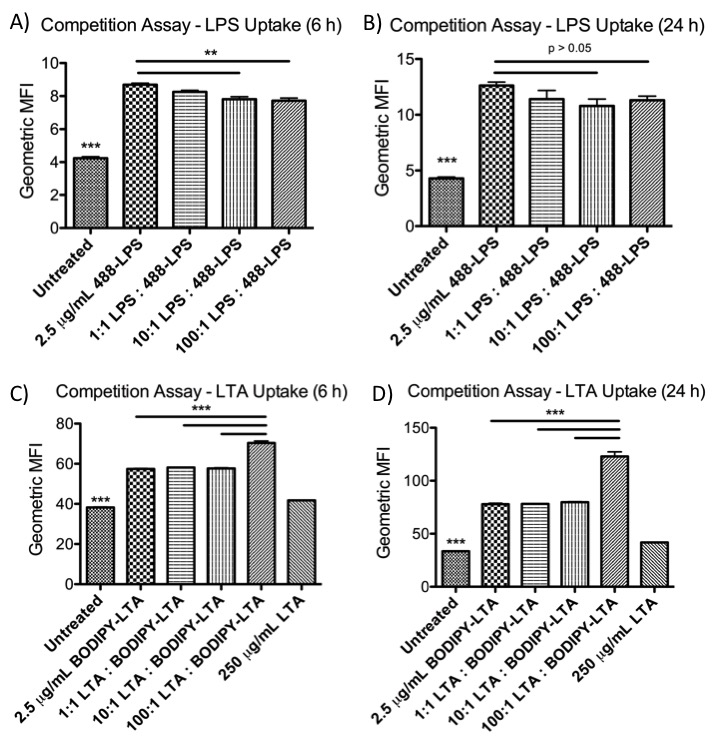


**Supplementary Fig. S5. Treatment with excess unlabeled LPS results in partial competitive inhibition of AlexaFluor 488-LPS uptake, whereas 100-fold excess of unlabeled LTA stimulates BODIPY 630/650-LTA uptake by HepG2 cells.** Cells were cultured in DMEM containing 20% normal serum and treated with 1:1, 10:1, and 100:1 ratios of unlabeled to fluorescently labeled (2.5 μg/mL concentrations of) LPS (A,B) or LTA (C,D) for either 6 h or 24 h. Flow cytometry was performed to measure the geometric mean fluorescence intensity (MFI) of 10,000 cells to quantify uptake of the fluorescently labeled LPS or LTA. Data are expressed as mean ± SEM, obtained from 3 experiments; **p<0.01, ***p<0.001.

**Supplementary Video S1.** **Dynamic live-cell confocal microscopy demonstrating increased LTA uptake over time.** Time-point #1 was taken 3.5 h post-treatment, and each subsequent time-point was taken every 30 min until 6 h post-treatment with 10 μg/mL BODIPY 630/650-LTA. Images are pseudo-coloured in green to enhance contrast of varying degrees of fluorescence intensity.

**Supplementary Video S2. Three-dimensional reconstruction of LTA uptake at 6 h using confocal microscopy.** Images are pseudo-coloured in green to enhance contrast of varying degrees of fluorescence intensity.
